# Rare case of complete colon structure in a mature cystic teratoma of the ovary in menopausal woman: a case report

**DOI:** 10.1186/s12905-016-0349-8

**Published:** 2016-10-28

**Authors:** Eun Young Ki, Dong Gyu Jang, Dong Jun Jeong, Chang Jin Kim, Sung Jong Lee

**Affiliations:** 1 Department of Obstetrics and Gynecology, Seoul St. Mary’s Hospital, College of Medicine, The Catholic University of Korea, Seoul, Korea; 2 St. Mary’s Women Hospital, Suwon, Korea; 3 Department of Pathology, College of Medicine, Soonchunhyang University, Cheonan, Korea; 4 Department of Obstetrics and Gynecology, St. Vincent Hospital, College of Medicine, The Catholic University of Korea, Suwon, Korea

**Keywords:** Neoplasms, Germ cell and embryonal, Teratoma, Ovary, Benign

## Abstract

**Background:**

Mature cystic teratoma (MCT) of the ovary is benign germ cell tumor and shows the highest incidence in women of reproductive age. Histologically, it includes components derived from endoderm, mesoderm, and ectoderm. Although there have been many reports of MCT having small part of the intestinal component, ovarian MCT containing complete colon structure was very rare.

**Case presentation:**

A 54-year-old woman underwent laparoscopic left salpingo-oophorectomy due to an incidentally found ovarian mass. The pathologic diagnosis of the ovary was MCT containing complete colonic structure. The colonic wall exhibited complete structure of the large intestine composed of mucosa, submucosa, proper muscle, subserosa and serosa. It also contained sebaceous gland, sweat glands, fat tissue, and bone. The patient recovered without any complications.

**Conclusion:**

Immunohistochemical staining can be used for differential diagnosis between MCT with colonic wall and mucinous tumor. We report a very rare case of MCT that had complete colon structure with a brief literature review.

## Background

Mature cystic teratoma (MCT) of the ovary, as a synonym for the ovarian dermoid cyst, is a benign germ cell tumor. The words “teratoma” and “dermoid” were first described by Leblanc in 1831 [1]. The incidence of MCT is 10–20 % of all ovarian tumors and 70 % of benign ovarian tumors in young women aged under 30 years. MCT shows the highest incidence in reproductive women (age range from 20 to 40 years) [2, 3]. MCT contains components originating from 3 germ cell layers (ectoderm, mesoderm, and endoderm) with different ratios, which include skin, neural components, teeth, cartilage, respiratory epithelium, and intestinal epithelium [3]. About 7–13 % of MCT cases include intestinal epithelium [4], however, there have been only a few cases of ovarian MCT including complete colon structures [5, 6].

Herein, we report an extremely rare case of MCT that had complete colon structures.

## Case presentation

**Fig. 1:**
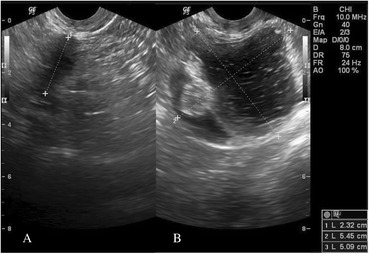
**a** Transvaginal ultrasonography of the right ovary shows no abnormal findings. **b** Transvaginal ultrasonography shows a left ovarian mass containing the solid portion representing a hyperechoic lesion and the fluid portion representing a hypoechoic lesion

**Fig. 2:**
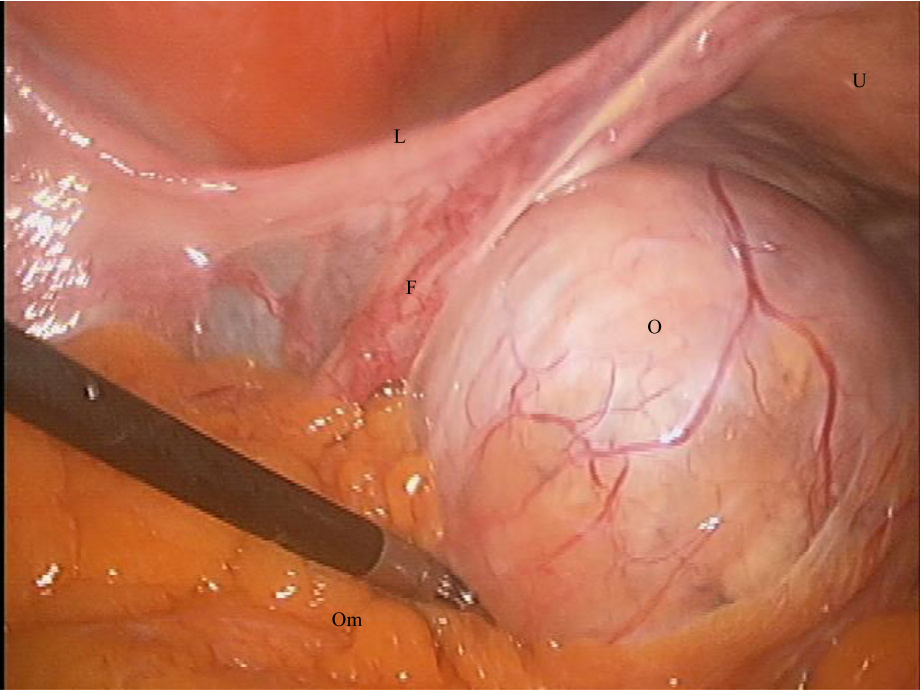
A left ovarian mass during operation. The surface is smooth, and the mass adheres to the omentum. L: Left round ligament, F: left fallopian tube, Om: omentum, O: Left ovary, U: uterus

**Fig. 3:**
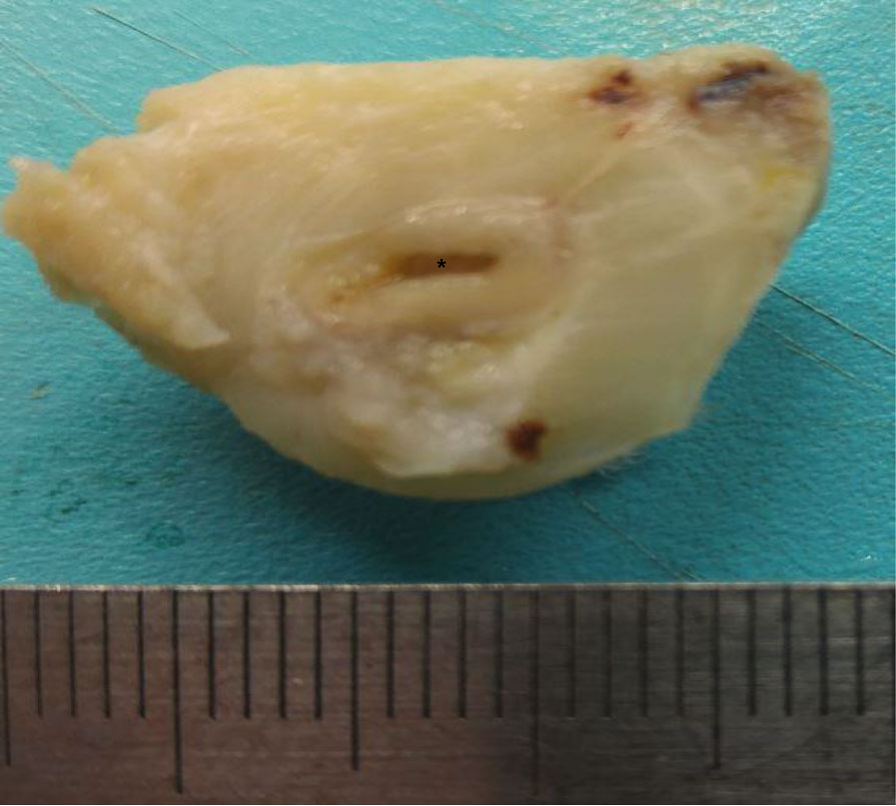
Macroscopic examination of the mass on the cystic ovarian inner surface. Sections of the oval round tissue reveal a lumen with muscle wall and smooth serosal surface (*)

A 54-year-old woman visited our outpatient clinic for pap-smear and routine medical check-up. She had a history of cesarean section and vaginal mesh operation due to urinary incontinence. She had no specific history of medication. We found a left ovarian cyst that had hypoechoic and hyperechoic lesions in the cyst on ultrasonography. The ovary measured 5.0 × 5.6 cm (Fig. 1). The right ovary and uterus showed no abnormal findings. Physical examination exhibited no tenderness or rebound tenderness on the left lower quadrant. The patient had negative cytologic findings on pap-smear. The patient underwent laparoscopic left salpingo-oophorectomy. The left ovarian mass tightly adhered to the omentum (Fig. 2), so we removed the left tube and ovary after adhesiolysis. Macroscopically, the ovarian mass was mainly filled with sebaceous materials, whose cystic wall contained complete colon structure (Fig. 3).

**Fig. 4:**
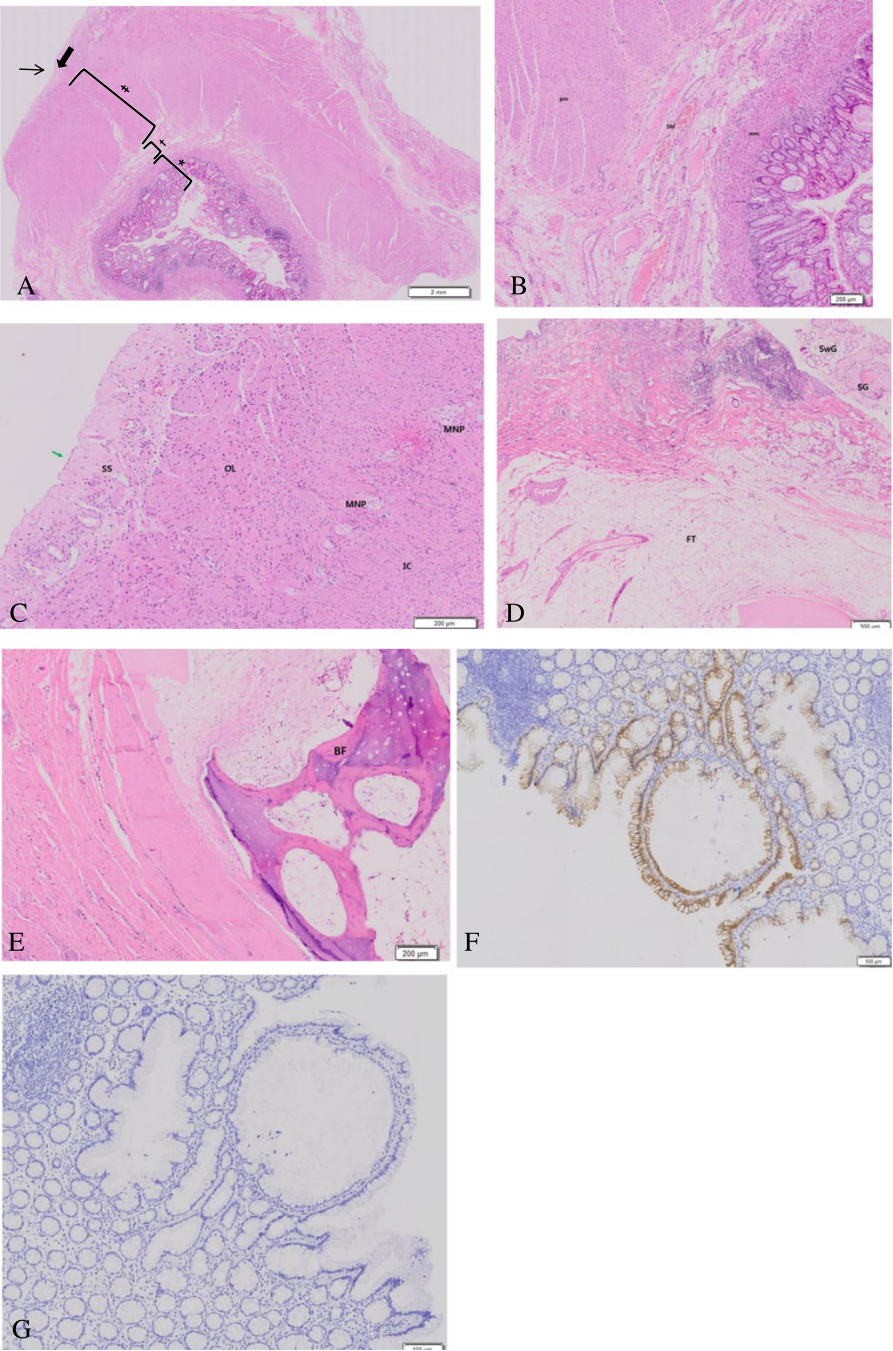
Microscopic findings of the ovarian mass. Sections of the oval round mass show **a** a complete structure of the large intestine composed of mucosa (*), submucosa (†), proper muscle (‡), subserosa (bold arrow) and serosa (thin arrow) (H&E). **b** Mucosa including mucosa muscle (mm), submucosa (sm) and proper muscle (pm) (H&E). **c** Proper muscle composed of inner circular (IC) and outer longitudinal (OL) layers, myenteric nerve plexus (MNP), subserosa (SS) and serosa (green arrow) (H&E). **d** A benign teratoma showing sebaceous glands (SG), sweat glands (SwG), and fat tissue (FT) (H&E). **e** The bone formation (BF) was also noted in the teratoma (H&E). Immunohistochemistry of colon mucosa in the ovarian mass reveals positivity for CK20 (**f**), and negativity for CK7 (**g**)

On light microscopy with hematoxylin and eosin staining, the pathologic diagnosis of the ovary was MCT containing complete colonic structures without any sign of malignancy (Fig. 4a). Normal colonic structure was composed of mucosa, submucosa, proper muscle, subserosa and serosa layer (Fig. 4a, b). The magnified view of the muscle layer exhibited well oriented inner circular and outer longitudinal muscle layers and myenterix nerve plexus (Fig. 4c). The ovarian mass also had sebaceous gland, sweat gland, fat tissue, and bone (Fig. 4d). It showed positivity for CK 20 and negativity for CK 7 on immunohistochemistry (Fig. 4f and g). These results supported the MCT containing colonic epithelial structure as normal. The patient recovered very well without complication.

## Discussion

MCT of the ovary can occur in women from adolescents to postmenopausal women. It usually develops unilaterally, but 8–15 % of MCT cases occur bilaterally. MCT is a slowly growing tumor, and the estimated increasing rate is 1.8 cm per year [7]. Furthermore, the long term recurrence rate is 4.7 % after surgery [8]. Therefore, fertility - sparing surgery has been performed on women with MCT in reproductive age [3].

Approximately 2 % of all MCT cases undergo malignant transformation. Squamous cell carcinoma is the most common type that account for 83 %, and adenocarcinoma, sarcoma, and carcinoid tumors account for the majority of the rest [3]. The intestinal type of MCT is associated with adenocarcinoma developed from MCT. Fishman et al. [9] documented a rare case of adenocarcinoma arising from gastrointestinal epithelium of MCT. Since then, many authors reported gastrointestinal epithelium associated with MCT [10–12]. As the germline components of MCT were arranged haphazardly, MCT containing well oriented complete intestinal structureis very rare, and only 3 cases have been reported in the literature (Table 1). Fujiwara et al. [5] reported 2 cases of MCT, one being a benign MCT containing a complete segment of the intestinal wall and the other being adenocarcinoma from MCT with complete colonic structure and bronchial epithelium as well. The prognosis of benign MCT was favorable, and patients were alive at 5 years after surgery without recurrences. Tang et al. [6] reported 1 case of a patient who was diagnosed with benign MCT containing complete colonic structures.

**Table 1:** Review of MCT containing complete colon structure or intestinal epithelium

Histology	Number	Age	Operation	Adjuvant treatment	Follow up	Authors
MCT with complete colon structure	1	35	USO	No	No document	Fujiwara et al. [5].
	2	45	TAH, BSO	No	NED for 5 years	
	3	16	USO	No	No document	Tang et al. [6].
	4	54	USO	No	No document	this study
MCT with adenocarcinoma of intestinal type	5	38	TAH, USO, omentectomy, appendectomy	5-FU, leucovorin	Death after 3 months	Fishman et al. [9].
	6	37	USO, PLND, PALND, omentectomy	No	NED for 40 months	Levin et al. [10].
	7	77	TAH, BSO, appendectomy	No	NED for 12 months	Min et al. [11].
	8	55	TAH, BSO, PLND, PALND, omentectomy	cisplatin and paclitaxel	NED for 6 months	Wheeler et al. [12].

*MCT* mature cystic teratoma, *TAH* total abdominal hysterectomy, *BSO* bilateral salpingo-oophorectomy, *USO* unilateral salpingo-oophorectomy, *PLND* pelvic lymph node dissection, *PALND* para-aortic lymph node dissection, *NED* no evidence of disease

Immunohistochemical staining can be used for differential diagnosis between MCT with colonic wall and mucinous tumor. They also identified that normal colons are positive for CK20 and negative for CK7 on immunohistochemistry. However, ovarian mucinous cystadenoma shows positivity for CK7 and negativity on CK 20 [6]. In contrast to benign mucinous epithelium, malignant epithelium associated with MCT more frequently shows negativity for CK7 and positivity for CK20, MUC2, and CDX2 [13]. Also, immunohistochemical positivity for CEA, CA19-9, and CK-20 was reported to have a strong relationship with malignant mucinous epithelium [9].

In our case, we found MCT of the ovary containing complete colonic structure in menopausal woman. Also, there was no evidence of malignant transformation.

## Conclusion

In summary, our case provides evidence that benign MCT of the ovary containing complete colonic structure shows positivity for CK 20 and negativity for CK 7 on immunohistochemistry.

## Abbreviations

MCT: Mature cystic teratoma
H & E: Hematoxylin and eosin
TAH: Total abdominal hysterectomy
BSO: Bilateral salpingo-oophorectomy
USO: Unilateral salpingo-oophorectomy
PLND: Pelvic lymph node dissection
PALND: Para-aortic lymph node dissection
NED: No evidence of disease
